# Young individuals with stroke: a cross sectional study of long-term disability associated with self-rated global health

**DOI:** 10.1186/1471-2377-14-20

**Published:** 2014-01-28

**Authors:** Susanne Palmcrantz, Lotta Widén Holmqvist, Disa K Sommerfeld

**Affiliations:** 1 Division of Physiotherapy, Department of Neurobiology, Care Sciences and Society, Karolinska Institutet, Huddinge, Stockholm, Sweden; 2 Division of Neurology, Department of Clinical Neuroscience, Karolinska Institutet, Stockholm, Sweden; 3 Department of Geriatric Medicine, Danderyd Hospital, Danderyd, Sweden; 4 Department of Physical Therapy, Karolinska University Hospital, Stockholm, Sweden

**Keywords:** Community, Disability, Health, Stroke, Young

## Abstract

**Background:**

Perceived disability after stroke may persist long-term even among young individuals with mild stroke and may be related to age-related expectations of health and recovery. Thus, in order to appreciate the magnitude of perceived disability in a younger stroke population studies are needed to explore perceived health-related differences between young individuals with stroke and a matched general population. Further, to provide long-term measures by health care, relevant to the same young individuals with stroke, their perceived long-term functioning and disability associated with health need to be explored.

**Methods:**

The generic questionnaire EQ-5D was used to compare ratings of global health and disability between young individuals living in the community up to 6 years after stroke (n = 150) and an age and geographically matched general population (n = 2661). Stroke related medical data were retrieved from medical records and the study specific questionnaire, the MYS-questionnaire, was used to assess self-rated disability associated with global health.

**Results:**

Among the young individuals 79% had suffered a mild stroke, 45% rated a low global health compared to 15% of the matched general population and a higher proportion rated problems in mobility, self-care, usual activities and anxiety/depression. Among the young individuals with stroke, limitations and restrictions in leisure activities, work, reading as well as low level of physical activity, utilizing personal care provider or personal assistance and tiredness were negatively associated with self-rated global health (R square 0.60).

**Conclusion:**

The negative effects of stroke, on self-rated global health among young individuals living in the community, appear to be substantial, multi factorial and long-standing which call for interdisciplinary research collaborations and team measures by health care long-term.

## Background

Health has been defined as comprising physical, mental and social aspects [1] as well as demands of life that commensurate with contextual factors such as age, culture and personal responsibility [2]. Among individuals with stroke, perceived disability involving various aspects included in these definitions of health, has been shown to persist long term after stroke [3-5]. In stroke populations, the International Classification of Functioning, Disability and Health (ICF) has been found to be favorable when used to map this diversity of aspects of functioning and disability associated with health [6]. The ICF has a body, individual and societal perspective and comprise various dimensions of health including the components body functions, body structures, activity, participation and contextual factors that are both personal and environmental [7]. Notably, in the total stroke population, individuals of working age (<65 years) are a minority and may deviate from the majority in expectations on functioning and health that commensurate with age related goals and expectations on recovery e.g. return to work [8-10]. These expectations may also be related to the nature of the national health and welfare system as well as the demands that are placed on the individual in their community and social life. Using a global rating of health, a recent Swedish study [5] finds that among individuals with stroke, disability in terms of limitations in mobility, and domestic life and mental impairments explain approximately half of the variance in self-rated health after stroke. However, in qualitative studies of individuals with stroke living in the community, younger individuals express more concerns about, e.g. returning to work while older individuals express difficulties more related to physical impairments, e.g. walking and difficulties in leaving the house, driving a car and using public transport [9,11,12]. Thus, in order to appreciate the magnitude of perceived disability in a younger stroke population, studies are needed to explore perceived health-related differences between young individuals with stroke and a matched general population. Further, to provide long-term measures by health care, relevant to the same young individuals with stroke, their perceived long-term functioning and disability associated with health need to be explored.

The aims of the study were to:

● explore differences between young individuals with stroke and a matched general population in long-term self-rated global health, functioning and disability

● explore aspects of long-term functioning and disability associated with self-rated global health in the same young individuals with stroke

## Methods

All young individuals of working age (18-64 years) were eligible for the study if they: 1) had been admitted to Södersjukhuset (Stockholm South General Hospital) Stockholm, Sweden, when suffering a stroke during 2000-2006, 2) had been registered in the Swedish Stroke Register (Riks-Stroke) [13], 3) were registered residents in the south of Stockholm and 4) were not institutionalized or living in sheltered accommodation at the time of the survey. Stroke related medical data in terms of stroke diagnosis according to the ICD-10 classification [14], stroke severity according to the Scandinavian stroke scale (SSS) classified as being mild, moderate or severe [15,16], lateralization, time for onset, sex, as well as diagnosed risk-factors for stroke (previous stroke, hypertension, hypercholesterolemia, cardiac arrhythmia, diabetes mellitus) were obtained from the hospital medical records.

In January 2007 a Swedish questionnaire consisting of 59 questions aiming to map young persons with stroke (the MYS questionnaire [17]) and the EQ-5D questionnaire [18] were distributed to 232 young individuals with stroke [19].

Prior to the present study the MYS questionnaire was constructed and tested in terms of content validity by an expert group and in terms of face validity, readability and stability by young individuals with stroke [17]. The MYS questionnaire was found to be valid thus, covering relevant aspects of functioning and disability among a selected sample of young individuals with stroke (n = 15) who were interviewed in a sampling to redundancy process [17]. The MYS-questionnaire was found to be stable (the test-retest agreement was overall fair to very good (kappa 0.44-1.0)) with the exception of 2 questions and 1 alternative answer which were not included in the present study [17]. Results on long-term health-states derived from the MYS questionnaire have previously been classified according to the components of the ICF and presented [19]. Included in the present study were:

1) impaired body functions (tiredness, memory, concentration, irritability, initiative, sleep, appetite, self-reported depression, stress, anxiety, bursting into tears in everyday situations, pain and impaired swallowing) were rated as impaired when experienced often/constantly and not impaired when experienced almost never/sometimes

2) limitations and restrictions in activity and participation (need of assistance in eating/drinking, toileting, caring for body parts, dressing, moving around indoors, moving around outdoors, using public transport, cooking, cleaning, shopping, economic transaction, and experienced limitations and restrictions in speaking, reading, writing, calculating, leisure activities and work) were rated as present or not.

3) personal factors (age, sex, educational level [ ≤ senior high school or university level education], smoking or not, low or moderate/high level of physical activity [with a cut off for low physical activity set at ≤ 1 hour of moderately strenuous activities/week]).

4) environmental factors (living alone, not experienced support from significant other, experienced dependence on significant other, receiving assistance from personal care provider or personal assistant, not receiving stroke related checkups by a physician, not experienced sufficient current rehabilitation) were rated as present or not.

EQ-5D is a generic measure used to assess health outcome and contains a quantitative measure that assesses perceived global health rated on a vertical, visual analogue scale (EQ VAS) which is calibrated with the anchors “worst imaginable health-state” (0) and “best imaginable health state” (100) [18]. Further, the EQ-5D includes 5 self-classifier health-state dimensions (EQ-5D dimensions): mobility, self-care, usual activities, pain/discomfort and anxiety/depression. Each EQ-5D dimension is graded according to the alternative statements: no problems, some problems or extreme problems. The measure is found to be valid and reliable and has been used in public surveys [18,20]. Evidence of construct validity has been found in comparisons with the SF-36 and the VAS has been found to be reliable in test retest (ICC 0.78) in general populations [18,20]. The EQ-5D has also been found to be valid and reliable in stroke-populations. In test of construct validity, increasing dysfunction reported with the EQ-5D domains has been found to be associated with lower scores on the standard instruments used to assess stroke outcome p < 0.0002 and in test-retest in a stroke population, a kappa ranging from 0.63 to 0.80 has been reported [21-23].

Comparative normative EQ-5D data were retrieved from an age (range) and geographically matched reference group in the general population [24]. This group had filled in the questionnaire approximately 6 months before the distribution of the EQ-5D questionnaires to the young individuals with stroke.

The study was approved by the Regional Ethical Review Board in Stockholm. Informed consent was obtained from the respondents to the questionnaires.

### Statistics

The t-test was used to assess differences between the young individuals with stroke and the matched general population with regard to self-rated global health (the EQ VAS). To determine the number of younger persons with stroke who rated a low global health a cut off was set by subtracting 1 SD from the mean ratings of global health in the matched general population. The alternative statements to the EQ-5D dimensions were dichotomized into no disability (no problems) and disability (moderate to severe problems). The Chi-squared test was used to analyze differences in the rated EQ-5D dimensions between the groups.

Four multiple linear regression analyses were performed using the following health-states from the MYS questionnaire as independent variables: 1) body functions and impairments, 2) activity, participation and limitations and restrictions, 3) personal factors and, 4) environmental factors. A 5^th^ multiple linear regression analysis was performed by entering all significant independent variables in analyses 1-4 in a final model. In each of the analyses the dependent variable was self-rated global health (the EQ VAS). In addition 1 multiple linear regression analysis was performed using the stroke related medical factors known at stroke onset (type, lateralization, mild or moderated/severe stroke and diagnosed risk factors for stroke) as independent variables. All analyses were controlled for sex and time since stroke onset. A significant level was set at p <0.05 and a stepwise method was used. An adjusted R-square explaining the variance by 0-0.25 was considered little to poor, 0.25-0.50 fair, 0.50-0.75 moderate and ≥0.75 very good to excellent [25].

## Results

Of the 232 young individuals with stroke 150 individuals (65%) filled in and returned the MYS and the EQ-5D questionnaires. The characteristics of the young individuals with stroke including stroke related medical factors and personal factors are presented in Table 1. The young individuals’ ratings of disability and reported environmental factors are presented in Table 2. A majority (79%) had suffered a mild stroke. Normative EQ-5D data was retrieved from 2661 geographically and age (range) matched individuals in the general population (median 46 years, inter quartile range 38-55 and 54% women). The young individuals with stroke rated significantly lower global health (mean 63, SD 24) than the matched general population (mean 79, SD 18) (p < 0.000). Forty-five percent (n = 67) of the young individuals with stroke as opposed to 15% (n = 392) in the general population rated low global health. The distribution of ratings in the EQ-5D dimensions is presented in Table 3. When differences between groups were explored significantly more individuals with stroke rated disability according to the EQ-5D dimensions: mobility, self-care, usual activities and anxiety/depression. No significant difference between groups was found in the EQ-5D dimension regarding pain/discomfort.

**Table 1:** Characteristics of the young individuals with stroke including stroke related medical factors and personal factors (n = 150)

	**n**	**%**
Age: median 59 years (IQR^a^ 54-62;); mean 57 years (SD^b^ 6); range 32-64	-	-
Women	50	33
Infarction/hemorrhage/not specified	120/26/4	80/17/3
Hemisphere: left/right/not specified	77/63/10	51/42/7
Stroke severity: mild/moderate/severe	119/19/12	79/13/8
Hypertension	69	46
Hypercholesterolemia	27	18
Previous stroke	26	17
Diabetes mellitus	21	14
Cardiac arrhythmia	e12	8
6 years^c^	19	13
5 years^c^	18	12
4 years^c^	15	10
3 years^c^	23	15
2 years^c^	22	14
1 year^c^	32	21
3 months to 1 year^c^	21	15
University level education	53	35
Smoking	49	33
Low level of physical activity ^d^	38	25

^a^Inter quartile range, ^b^Standard deviation ^c^Years since stroke onset ^d^ ≤ 1 hour of moderately strenuous activities/week.

**Table 2:** The number of young individuals with rated disability and reported environmental factors (n = 150)

**Disability**	**n**	**%**
*Impairment*		
Tiredness	67	45
Concentration	34	23
Irritability	32	21
Memory	30	20
Initiative	30	20
Pain	28	19
Stress	28	19
Anxiety	27	18
Self-reported depression	27	18
Sleep	26	17
Bursting into tears in everyday situations	15	10
Impaired swallowing	10	7
Appetite	8	5
*Activity limitation and participation restriction*		
Leisure activities	85	57
Work	59	39
Writing	46	31
Cleaning	40	27
Shopping	37	25
Economic transaction	35	23
Reading	33	22
Cooking	29	19
Speaking	27	18
Calculating	27	18
Using public transport	17	11
Moving around outdoors	16	11
Caring for body parts	13	9
Dressing	13	9
Moving around indoors	4	3
Eating/drinking	3	2
Toileting	3	2
**Environmental factors**	**n**	**%**
Not receiving stroke related checkups	74	50
Living alone	65	43
Dependence on significant other	51	34
No support from significant other	39	26
Not sufficient current rehabilitation	39	26
Receiving assistance from personal care provider or personal assistant	24	16

**Table 3:** The distribution of individual ratings according to the EQ-5D self-classifier dimensions presented for young individuals with stroke (YWS) (n = 150) and an age and geographically matched general population (GP) (n = 2661)

**Dimension**	**YWS%**	**GP%**	**p**	**YWS women**	**GP women**	**p**	**YWS men**	**GP men**	**p**
Mobility									
No problem	60	91		64	91		57	91	
Moderate/severe problem	40	9		36	9		43	9	
			<0.0000			0.0004			<0.0000
Self care									
No problem	83	98		85	98		82	97	
Moderate/severe problem	17	2		15	2		18	3	
			0.0003			0.0010			0.0005
Usual activities									
No problem	72	90		73	89		71	91	
Moderate/severe problem	28	10		27	11		29	9	
			0.0012			0.0039			0.0003
Pain/discomfort									
No problem	48	56		44	52		50	60	
Moderate/severe problem	52	44		56	48		50	40	
			0.2575			0.2575			0.1552
Anxiety/depression									
No problem	47	64		38	61		51	67	
Moderate/severe problem	53	36		62	39		49	33	
			0.0156			0.0011			0.0214

Results of the 5 linear multiple regression analyses that explored the association between self-rated functioning and disability (assessed with the MYS questionnaire) and self-rated global health (EQ VAS) among the younger individuals with stroke are presented in Table 4. Sex and time since stroke onset were not associated with self-rated global health in any of the analyses. In the final model, health-states from the MYS questionnaire explained 60% of the variance in self rated global health. The main contributing factors were limitations and restrictions in return to previous leisure activities and not having returned to work which were negatively associated with self-rated global health.

**Table 4:** Results of the 5 linear multiple regression analyses exploring self-rated functioning and disability (assessed with the MYS questionnaire) that were associated with self-rated global health (EQ VAS) among the younger individuals with stroke

	**B**^**a**^	**95% CI**^**b**^		**p**	**Adjusted R square**
1. Impairments					
Tiredness	-12.3	-19.6	-5.0	0.001	
Initiative	-11.5	-20.4	-2.7	0.011	
Depression	-12.2	-21.5	-2.9	0.010	
Pain	-10.2	-19.1	-1.3	0.026	
					0.32
2. Activity limitations and Participation restrictions					
Leisure activities	-15.6	-21.9	-9.3	0.000	
Work	-11.3	-17.5	-5.1	0.000	
Reading	-14.0	-21.6	-6.4	0.000	
Cleaning	-11.0	-18.1	-3.9	0.003	
					0.50
3. Personal factors					
Low physical activity	-21.2	-29.6	-12.9	0.000	
					0.15
4. Environmental factors					
Dependence on significant other	-18.2	-25.6	-10.7	0.000	
Personal assistance^c^	-20.2	-30.1	-10.3	0.000	
					0.30
5. Final model^d^					
Leisure activities	-14.3	-20.5	-8.2	0.000	
Work	-11.7	-17.5	-6.0	0.000	
Reading	-10.2	-17.6	-2.7	0.008	
Low physical activity	-10.8	-16.8	-4.9	0.000	
Personal assistance^c^	-10.8	-18.8	-2.9	0.008	
Tiredness	-7.6	-13.6	-1.6	0.013	
					0.60

The independent variables which were the main contributors to explain the variance in self-rated global recovery are presented first and thereafter in descending for each analysis.

^a^Regression coefficient, ^b^Confidence Interval, ^c^Utilizing personal care provider or personal assistance, ^d^Including all significant independent variables in models 1-4.

In the multiple linear regression analysis including stroke related medical factors known at stroke onset, only stroke severity was associated with self-rated global health (B -19.254, CI 95% -28.553 to -9.956, p < 0.000, Adjusted R square 0.10) rated 3 months to 6 years after stroke onset.

## Discussion

This study found lower ratings of health among young individuals with stroke compared to a matched general population in Sweden. Although 79% of the young individuals had suffered a mild stroke, 45% rated a low global health compared to the matched general population. Thus, in this younger stroke population in Sweden, a substantial negative impact of stroke on self-rated health was found. In addition, with a moderate explanatory level of 60% and supported by the literature presented below, we may conclude that relevant multi factorial aspects that negatively influence global health long-term were targeted. These aspects included limitations and restrictions in leisure activities and work, negatively associated with global health and have previously been found to be the most commonly reported aspects of disability in young individuals with stroke [19,26]. Performing leisure activities has been found to be a way of coping among young individuals after stroke [27] and not being able to return to work has been found to be associated with lower ratings of physical health [28]. Thus, return to leisure activities and work need to be in focus in rehabilitation planning. Factors influencing return to work involves not only impairments following stroke but also personal and environmental factors emphasizing the need for individualized measures [29]. Nonetheless, the beneficial effect of vocational rehabilitation has yet to be established thus further research is needed [30]. Moreover, limitation and restriction in reading was negatively associated with global health. Reading difficulties after stroke may be due to impaired mental functions such as impaired memory, concentration or language functions. Additionally, visual impairments have been found to be a common cause of reading difficulties and are treatable with assistive devices [31]. Thus, visual impairments should not be neglected in long-term assessments by health-care. Further, low physical activity was negatively associated with global health. Lack of information about, and fear of physical exertion have been reported among young individuals with stroke [32], and a dose response between physical activity and cardiovascular disease has been found [33]. Thus physical activity is a matter that needs to be targeted in long-term health care measures after stroke [34]. The negative association between utilization of a personal care provider or a personal assistant and self-rated global health found in the present study may be due to experienced lack of independence [35]. However, dissatisfaction with the assistance given may also be involved [36]. The effects of stroke are multi factorial which points to the necessity of stroke specific training for personal care providers and assistants.

Furthermore, tiredness was negatively associated with global health. Tiredness experienced often or constantly has been commonly reported [19]. Moreover, tiredness defined as fatigue has been found to be associated with disability and low ratings of general health among young individuals with stroke [37]. Further, as fatigue has been found to be negatively associated with return to work after stroke [38], we may assume that fatigue is a major concern long-term. A recent study demonstrates promising results in treating long-term fatigue by means of cognitive and graded physical activity training as well as by teaching compensation strategies [39]. Treatment for stroke related fatigue is a matter that needs to be explored in future studies.

This study has thus identified relevant aspects of global health that need to be targeted when long-term measures are allocated by the health-care system.

As more than 80% return home after stroke in Sweden [40], the young individuals in the present study who were all living in the community after stroke may be considered representative to young individuals with stroke. However, the study sample was limited and restricted to the capital of Sweden thus the representativeness may be questioned. Further, the results of the multiple linear regression analyses need to be interpreted with caution due to the width of the confidence intervals. Still, our findings, supported by the literature, indicate that presented health-states that were negatively associated with self-rated global health are long standing and commonly reported.

Notably none of the diagnosed risk factors were significantly associated with long-term self-rated global health. Further, although significantly associated, stroke severity at onset, poorly explained the variance in self-rated global health 3 months to 6 years after stroke. Instead, the long-term effects of stroke relevant to the young individuals often appear to be oriented toward mental impairments and activity limitations and participation restrictions [27,41,42]. Further, as a result of the validation process of the MYS questionnaire in which young individuals with stroke took part, the MYS questionnaire focuses on mental impairments and a majority of the questions deal with activity limitations and participation restrictions [17]. However, other aspects of disability have been reported as relevant among young individuals after stroke such as not being able to run a shorter distance [32] and depression of sexual activity [43]. These aspects could be considered in future studies of factors associated with self-rated global health.

We found no difference in the pain/discomfort dimension between the young individuals with stroke and the matched general population. Furthermore, similar ratings of pain/discomfort have been reported in a Swedish national survey including young individuals [20]. Notably, the occurrence of rated pain in the general population has been found to account for only half of the occurrence of rated pain/discomfort [44]. This finding indicates that there is a great difference between rated pain and rated pain/discomfort. Thus, future studies need to consider what these ratings of pain/discomfort incorporates as well as how the expression “discomfort” is interpreted by the rater.

## Conclusion

The negative effects of stroke on self-rated global health found in the present study were substantial, long-standing and multi factorial among the young individuals with stroke living in the community. This disparity of factors that influence self-rated global health long-term reveals a need of multi professional assessments and measures by health-care in a long-term perspective, irrespective of initial stroke severity.

## Competing interests

The authors declare that they have no competing interests.

## Authors’ contributions

SP performed the statistical analyses and is the first author. SP, LW and DS have made substantial contributions to design, analysis and interpretation of the data and LW and DS in commenting on the drafted manuscript. All authors read and approved the final manuscript.

## Pre-publication history

The pre-publication history for this paper can be accessed here:

http://www.biomedcentral.com/1471-2377/14/20/prepub
